# Adverse Childhood Experiences Are Associated With Adult Dream Content: A Cross-Sectional Survey

**DOI:** 10.3389/fpsyg.2022.837347

**Published:** 2022-04-08

**Authors:** Yundong Ma, Xia Feng, Di Wang, Xiaoxia Zhao, Zejun Yan, Yanping Bao, Ran Zhu, Qiqing Sun, Jiahui Deng, Lin Lu, Hongqiang Sun

**Affiliations:** ^1^NHC Key Laboratory of Mental Health (Peking University), Peking University Sixth Hospital, National Clinical Research Center for Mental Disorders (Peking University Sixth Hospital), Peking University Institute of Mental Health, Beijing, China; ^2^The Second People’s Hospital of Guizhou Province, Guiyang, China; ^3^Beijing Changping Hospital of Integrated Chinese and Western Medicine, Beijing, China; ^4^School of Biomedical Science and Medical Engineering, Beihang University, Beijing, China; ^5^National Institute on Drug Dependence and Beijing Key Laboratory of Drug Dependence, Peking University, Beijing, China; ^6^School of Public Health, Peking University, Beijing, China; ^7^Peking-Tsinghua Center for Life Sciences and PKU-IDG/McGovern Institute for Brain Research, Peking University, Beijing, China

**Keywords:** childhood adversities, emotional neglect, dream recall, dream content, Hall and Van de castle coding system

## Abstract

**Background:**

Dreams can be affected by recent life events and long-term life experiences. Previous evidence has shown that childhood adverse experiences are associated with sleep quality and dream experiences.

**Objective:**

The aim of this study was to explore the relationship between childhood adverse experiences and dream content in adults.

**Participants and Setting:**

A total of 163 participants without current or past physical or mental disorders aged between 18 and 35 were screened in the hospital. Among them, 120 subjects who completed a dream content record at home and whose anxiety and depression levels and sleep quality were within the normal range were included in the data analysis.

**Methods:**

A cross-sectional survey was conducted from June 2017 to December 2019. Dream content for 10 consecutive days was recorded by the participants and coded by the Hall and Van de Castle coding system. Childhood adversity was assessed by the Childhood Trauma Questionnaire (CTQ). In the end, 719 dreams out of 626 nights for 120 participants (44 female) were included in the data analysis, gender differences between groups were analyzed using *t*-tests or *U* tests, and Spearman’s partial correlation and multiple linear regression were used to investigate the relationship between childhood trauma and dream content.

**Results:**

Childhood adversity was associated with characters, friendly interactions, and objects in dream content. Regression models of childhood adversity predicting characters and objects in dream content were constructed. There were no gender differences in general demographic data, sleep quality, emotional state, childhood adversity, dream recall frequency, or dream content.

**Conclusion:**

Childhood adversity is associated with adult dream content.

## Introduction

A dream is a basic physiological phenomenon that often appears during sleep and includes, for example, thoughts, emotions, perceptions, and images (Pagel et al., 2001). Dreams can also be conceived as experience of consciousness or phenomenal states during sleep (Windt, 2015). In addition, dreams are considered proof of the speculation that consciousness is encapsulated within the brain (Revonsuo, 2006). Dreaming is the subjective process of experiencing these mental activities. Among normal adults, 93.5% have nightly dream memories and 6.5% have almost no dream memories. (Pagel, 2003) Additionally, adults reported approximately 1–2.8 dream memories per week in a dream questionnaire (Schredl, 2008; Nielsen, 2012) and 2.38 dream recalls per week when a home dream diary was completed (Goodenough, 1991). Dream content may be influenced by sociodemographic factors and health status. Thematic diversity in dream content has been reported to decrease with age, and the prevalence of disturbing dreams in young and middle-aged people is higher than that in older adults (Worley et al., 2021). Patients with nightmare disorder, rapid eye movement sleep behavior disorder, and posttraumatic stress disorder have more negative emotional content in their dreams (Phelps et al., 2018; Gieselmann et al., 2019; Antelmi et al., 2021; Stefani and Hogl, 2021).

Daytime life and childhood experiences have an impact on dream content. Dreamers’ life experiences within the week before they dream may reappear in dreams (Picard-Deland and Nielsen, 2021). Childhood adversities refer to situations or experiences that create difficulty or unpleasantness for an individual before the age of 16 (Bernstein et al., 2003). According to the WHO World Mental Health Surveys, the prevalence of exposure to at least one childhood adversity (including interpersonal loss, parental maladjustment, maltreatment, physical illness, and economic adversity) is approximately 38.8% worldwide, irrespective of a country’s level of economic development. Of those who have reported any adverse childhood event, approximately 60% have been exposed to multiple adversities (Kessler et al., 2010; Morgan and Gayer-Anderson, 2016). Childhood adversity is a well-documented risk factor for the development of clinical or subclinical psychosis in adolescence and adults (Varese et al., 2012; Rossler et al., 2014). Childhood adversity has also been linked to ultrahigh-risk status and more severe disease processes and outcomes, such as an increased risk of substance abuse, earlier illness onset, and a higher risk of comorbidity (Arseneault et al., 2011; Agnew-Blais and Danese, 2016; Hunt et al., 2017; Gaweda et al., 2020). Severe emotional abuse (EA), emotional neglect (EN), physical neglect (PN), and sexual abuse (SA) have been associated with suicidal ideation and attempts (de Araujo and Lara, 2016).

Evidence has demonstrated that childhood adversities are associated with sleep problems in adolescence (April-Sanders et al., 2021). Children who suffer from interpersonal violence, such as parent to child violence, rape, or trauma, including accidents or injuries, show a higher risk of sleep disturbance and sleep disorders (Kajeepeta et al., 2015; Wang et al., 2016). Individuals with a history of emotional and physical abuse during childhood have poorer sleep quality in adulthood (Ramsawh et al., 2011). Childhood adversity may also influence dreams, and children who are separated from their mothers before 1 year of age have more nightmare experiences as adults (Csoka et al., 2011; Nielsen et al., 2019). There is also a correlation between attachment style and dream content that dreams may promote attachment in insecurely attached adults (McNamara, 1996). Compared with those classified as securely, avoidant, or dismissing attachment styles, individuals classified as preoccupied attachment style are more likely to have dreams and describe their dreams more loquaciously (McNamara et al., 2001). Anxious and avoidant attachment are associated with high levels of stress and conflict in dreams, and individuals with anxious and avoidant attachment have higher levels of anxiety and jealousy in dreams (Selterman and Drigotas, 2009). However, the relationship between childhood adversities and dream content in adulthood is not yet fully understood.

In this study, we hypothesized that adult dream content is influenced by childhood adversity. We sought to encode and quantify dream content using the text version of the Hall and Van de Castle (HVDC) coding system of dream content (Hall et al., 1966).

## Materials and Methods

### Participants

A total of 163 participants aged between 18 and 35 years without a history or family history of mental health disorders were screened between June 2017 and December 2019. For the screening evaluation, all participants were interviewed regarding their history and status of physical and mental disorders, assessed using the Mini-International Neuropsychiatric Interview (MINI)-4 (Sheehan et al., 1998). The participants were asked to record their dreams in writing immediately after they awoke from a dream over the next 10 days. Signed informed consent was obtained from all participants before the experiment, and ethical approval for the study was obtained from the Ethics Committee of the Peking University Sixth Hospital.

### Measures

Demographic data (i.e., age, gender, ethnicity, residence, educational level, and dream recall frequency) were collected. Depressive and anxiety symptoms and sleep quality were assessed using the Zung Self-Rating Depression Scale (SDS), Zung Self-Rating Anxiety Scale (SAS), and Pittsburgh Sleep Quality Index (PSQI), respectively. The severity of childhood adversities was assessed using the Chinese version of the Childhood Trauma questionnaire-28 item Short Form (CTQ-SF). Because our study focused on dream content in healthy adults, participants who presented with abnormal levels of anxiety or depression, or sleep quality problems were excluded. The SDS and SAS are self-rating scales, of which both have 20 questions scored from 1 to 4 (none or a little of the time, some of the time, a good part of the time, and most of the time, respectively). There are 10 questions each concerning increasing and decreasing depression levels in SDS, respectively (Zung, 1972). Participants with SDS scores less than 50 were included in the analysis. There were fifteen positively worded and five negatively worded questions in SAS (Zung, 1971). Participants with SAS scores that less than 45 were included in the analysis. The PSQI is a self-rated questionnaire that is widely used to assess sleep quality within 1 month. The PSQI includes 19 self-rated and five other-rated questions that can be further categorized into seven subscales (sleep duration, sleep latency, sleep disturbances, sleep quality, sleep efficiency, daytime dysfunction, and use of sleep medication). Participants who scored less than or equal to 5 on the PSQI were included in the analysis (Buysse et al., 1989). The CTQ-SF is a self-report questionnaire used to assess childhood adversity before the age of 16 years. It comprises 28 items generating a total trauma score and has five subscales: emotional abuse (EA), physical abuse (PA), sexual abuse (SA), physical neglect (PN), and emotional neglect (EN). Responses are recorded using a five-point Likert scale ranging from 1 (never true), 2 (rarely true), 3 (sometimes true), 4 (often true), to 5 (very often true; Bernstein et al., 2003). We analyzed childhood trauma data as continuous variables. Finally, 43 of the 163 participants were excluded because they exceeded the cutoff score on the SAS, SDS, or PSQI, or because CTQ-SF data were missing.

Participants were asked to record their dreams in as much detail as possible immediately after waking up in the morning for 10 consecutive days. The introductory words on the dream record sheet are as follows: Please answer whether you were dreaming, and if there are dreams with content, please describe the content of your dreams in detail. We then counted the number of days involving dreaming, the total number of dreams recalled, and the total number of words in the dream content descriptions of the participants. Because some participants had more than one dream recalled on a particular day, we calculated the number of dreams recalled on the days when at least one dream was recalled. Ultimately, 719 dreams out of 626 nights for 120 participants (44 women) were included in the data analysis.

Dream content was analyzed using an HVDC coding system (Hall et al., 1966). This is the most commonly used empirical system for analyzing dream content because of its comprehensiveness. This scale has several nominal categories within dream reports: characters, social interactions, activities, success and failure, misfortune and good fortune, emotions, settings, objects, and descriptive elements (Domhoff and Schneider, 1998; Domhoff, 2003). Two judges, who were blinded to the groups and adequately trained in the use of the scale, scored these dreams. After the judges reached a consensus on the scores by consulting, they entered the scores into DreamSAT, which is a spreadsheet that facilitates the statistical analysis of dreams coded with the Hall/Van de Castle system of quantitative content analysis. More details of DreamSAT can be found on the website of Dream Bank http://www.dreamresearch.net/ (Schneider and Domhoff, 2022). Owing to the differences in the number of words and elements recorded by each person in the contents of dreams, and to reduce the bias caused by the differences, it is also necessary to explore the possibility of whether the proportion of each element in dreams change with other variables. We calculated the proportion of all categories based on the total number of categories and statistically analyzed the proportions as independent variables. To reflect the stability of the subjects over time, the data of all the dream contents recorded by each subject during these 10 days were analyzed as a whole, and not individually.

### Statistical Analysis

Statistical analysis was performed using IBM SPSS Statistics version 24. Kolmogorov–Smirnov tests were used to test the normality of the distribution of the data, including demographic data, emotional state, sleep quality, dream memory, and dream content. Two-sample *t*-tests on data that conformed to the normal distribution were used to determine the differences between the sexes, and *Mann–Whitney U* tests were used to analyze the data that were no normally distributed. Data that conformed to a normal distribution are presented as mean ± standard deviation (SD), and data that did not conform to a normal distribution are presented as median (interquartile range). As we did not find gender differences in dream content, we combined data from all genders in subsequent analyses.

Correlations between age, years of education, anxiety level, depression level, sleep quality, and dream content were tested using *Spearman’s correlations*. The relationship between total childhood trauma scores and dream content was analyzed using *Spearman’s partial correlations*, with age, years of education, and SAS, SDS, and PSQI scores as covering variables to exclude the effects of current mood and sleep states on CTQ scores and dream content relationships. We then explored which specific negative experiences on the CTQ were associated with particular categories, and *Spearman’s partial correlations* were also based on age, years of education, and SAS, SDS, and PSQI scores as covariates.

We performed *multiple linear regression* tests to construct prediction models for the proportions of various elements in dream content. The dependent variables in the model are those elements of the dream contents that have positive results in *Spearman’s correlations* or *Spearman’s partial correlations*, and the independent variables were the demographic data or CTQ questionnaire scores on the side of the correlation with the dependent variables in the previous analysis.

## Results

### Demographic Information

The demographic characteristics, dream recalls, and dream contents of all participants are shown in Table 1. This study included 120 subjects, including 44 women, with a median age of 25 years and a median education level of 17 years. In this 10-day dream report, the median number of dreaming days for men and women was 5. The median numbers of dreams in men and women were 5 and 6, respectively. There were no significant differences across genders in the general demographic data, anxiety levels, depression levels, sleep quality, frequency of dream memories, or dream contents across categories.

**Table 1:** Demographic characteristics and dream recall and dream contents.

	**Male (*N* = 76)**	**Female (*N* = 44)**	**t/Z**	**Value of *p***
Age (Y)	25 (22, 26.75)	25 (23, 26)	−0.592	0.554
Education (Y)	17 (16, 19)	17 (16, 19)	−0.397	0.692
SDS	32 (28, 36)	33 (29, 37)	−0.516	0.606
SAS	30.5 (27, 33)	31 (28, 33)	−0.049	0.961
PSQI	2.5 (2, 4)	3 (2, 4)	−0.778	0.436
CTQ	29 (26, 32)	28 (27, 32)	−0.323	0.747
EA	5 (5, 7)	6 (5, 7)	−0.821	0.412
PA	5 (5, 5.75)	5 (5, 5)	−1.555	0.120
SA	5 (5, 5)	5 (5, 5)	−1.263	0.207
EN	7 (5, 9)	6 (5, 8.75)	−0.497	0.619
PN	5 (5, 7)	5 (5, 6)	−0.251	0.802
Dreaming days	5 (3.25, 7)	5 (4, 7)	−1.296	0.195
No. of dreams	5 (4, 7)	6 (4, 8)	−1.454	0.146
Dreams on dreaming days	1 (1, 1.38)	1 (1, 1.24)	−1.389	0.165
Word count	326.50 (200.25, 646.25)	466.50 (293.25, 784.50)	−1.639	0.101
Unique elements	31.00 (52.00, 89.75)	71.00 (39.50, 109.75)	−1.808	0.071
Characters	14.63% (10.29, 19.48%)	14.56% (11.96, 19.85%)	−0.852	0.394
Aggression	2.70% (00.00, 04.87%)	2.26% (00.21, 03.75%)	−0.826	0.409
Friendliness	1.59% (0.00, 2.76%)	2.15% (0.32, 3.24%)	−1.768	0.077
Sexuality	0.00% (0.00, 0.00%)	0.00% (0.00, 0.00%)	−0.142	0.887
Activities	16.69% ± 6.76%	17.57% ± 5.49%	−0.735	0.464
Failure	0.00% (0.00, 0.00%)	0.00% (0.00, 0.00%)	−0.919	0.358
Success	0.00% (0.00, 0.00%)	0.00% (0.00, 0.00%)	−1.307	0.191
Misfortune	1.63% (0.00, 3.24%)	1.44% (0.00, 2.56%)	−0.584	0.559
Good fortune	0.00% (0.00, 1.01%)	0.00% (0.00, 0.00%)	−1.251	0.211
Emotions	2.84% (1.13, 5.56%)	2.37% (0.00, 4.19%)	−1.685	0.092
Settings	11.22% (8.23, 15.71%)	10.51% (7.68, 12.19%)	−1.699	0.089
Objects	22.69% ± 8.78%	23.94% ± 7.47%	−0.795	0.428
Descriptive elements	20.86% ± 7.49%	21.50% ± 7.63%	−0.446	0.656

SDS, Zung Self-Rating Depression Scale; SAS, Zung Self-Rating Anxiety Scale; PSQI, Pittsburgh Sleep Quality Index; CTQ, Childhood Trauma Questionnaire; EA, emotional abuse; PA, physical abuse; SA, sexual abuse; PN, physical neglect; and EN, emotional neglect. The data that conformed to a normal distribution are presented as the mean ± standard deviation (SD), and the data that did not conform to a normal distribution are presented as the median (interquartile range). Dream content characteristics were calculated as proportions.

### Correlations Between Demographic Characteristics and Dream Content Assessed as Proportions

Correlations between demographic data, anxiety and depression levels, dream recall, and dream content were shown in Table 2. As age increased, the proportion of sexual interactions (*r* = 0.220, *p* = 0.016) and good fortune (*r* = 0.185, *p* = 0.043) in the dream content increased. Increased depression levels were associated with an increased proportion of dreams involving aggressive interactions (*r* = 0.264, *p* = 0.004). However, with higher SAS scores, there was a greater proportion of aggressive interactions (*r* = 0.210, *p* = 0.021) and a lower proportion of descriptive elements (*r* = −0.211, *p* = 0.021). Poor sleep quality was associated with a higher proportion of characters in dream content (*r* = 0.182, *p* = 0.047).

**Table 2:** Correlations between demographic characteristics, anxiety and depression levels, and sleep qualities and characters, aggression interactions, sex interactions, good fortune, and descriptive elements in the dream content.

	Characters	Aggression interactions	Sex interactions	Good fortune	Descriptive elements
Age (Y)	–	–	*r* = 0.220 *p* = 0.016	*r* = 0.185 *p* = 0.043	–
Education (Y)	–	–	*r* = 0.185 *p* = 0.043	–	–
SDS	–	*r* = 0.264 *p* = 0.004	–	–	–
SAS	–	*r* = 0.210 *p* = 0.021	–	–	*r* = −0.211 *p* = 0.021
PSQI	*r* = 0.182 *p* = 0.047	–	–	–	–

SDS, Zung Self-Rating Depression Scale; SAS, Zung Self-Rating Anxiety Scale; and PSQI, Pittsburgh Sleep Quality Index. Dream content characteristics were calculated as proportions.

Participants with higher total childhood trauma scores had a lower proportion of life-like characters (*r* = −0.186, *p* = 0.047) and a lower proportion of friendly interactions between characters (*r* = −0.212, *p* = 0.023) but an increase in the proportion of dreams with inanimate objects (*r* = 0.216, *p* = 0.020). Of all the subscales of the CTQ, only emotional neglect was associated with the above three categories. As the emotional neglect scores increased, there was a lower proportion of participants’ dreams with life-like characters (*r* = −0.251, *p* = 0.007) and friendly interactions between characters (*r* = −0.217, *p* = 0.020), but an increase in the proportion of dreams involving inanimate objects (*r* = 0.299, *p* = 0.001). The correlations between the total childhood trauma scores, emotional neglect scores, and dream content were shown in Table 3.

**Table 3:** Correlations between total childhood trauma and emotional neglect scores and characters, friendly interactions, and objects in the dream content.

	Characters	Friendly Interactions	Objects
CTQ	*r* = −0.186 *p* = 0.047	*r* = −0.212 *p* = 0.023	*r* = 0.216 *p* = 0.020
EN	*r* = −0.251 *p* = 0.007	*r* = −0.217 *p* = 0.020	*r* = 0.299 *p* = 0.001

CTQ, Childhood Trauma Questionnaire; EN, emotional neglect. Dream content characteristics were calculated as proportions.

### Predictive Models of the Content of Dreams

We constructed a multilinear regression model for the dependent variables based on dream content that was related to two or more indicators, such as demographic data, anxiety and depression levels, sleep quality, and CTQ scores. First, we used PSQI, total childhood trauma, and emotional neglect scores as independent variables; the proportion of dreams including characters as the dependent variable; and the method chosen used a backward selection procedure. This model was significant (*R*^2^ = 0.069, *F* = 4.358, *p* = 0.015), with a significant contribution from the PSQI scores (*B* = 0.009, *t* = 2.296, *p* = 0.023), emotional neglect scores (*B* = −0.004, *t* = −2.086, *p* = 0.039), and the intercept of this model (*B* = 0.159, *t* = 9.722, *p* < 0.001; see Table 4).

**Table 4:** A multiple linear regression model with characters as the dependent variable.

	*B*	*95%CI of B*	*SE*	*t*	*p*	*R*	*R* ^2^	*F*	*p*
Intercept	0.159	0.126, 0.191	0.016	9.722	<0.001	0.263	0.069	4.358	0.015
PSQI	0.009	0.001, 0.016	0.004	2.296	0.023				
EN	−0.004	−0.007, −0.0002	0.002	−2.086	0.039				

PSQI, Pittsburgh Sleep Quality Index; EN, emotional neglect. Dream content characteristics were calculated as proportions.

We then built a model with the proportion of dreams involving objects as the dependent variable, which was also statistically significant (*R*^2^ = 0.073, *F* = 9.316, *p* = 0.003), in which the total childhood trauma scores contributed (*B* = 0.004, *t* = 3.052, *p* = 0.003), as did that the intercept (*B* = 0.109, *t* = 2.655, *p* = 0.009; see Table 5).

**Table 5:** A multiple linear regression model with objects as the dependent variable.

	*B*	*95%CI of B*	*SE*	*t*	*p*	*R*	*R* ^2^	*F*	*p*
Intercept	0.109	0.028, 0.190	0.041	2.655	0.009	0.271	0.073	9.316	0.003
CTQ	0.004	0.001, 0.007	0.001	3.052	0.003				

CTQ, Childhood Trauma Questionnaire. Dream content characteristics were calculated as proportions.

## Discussion

This study illustrated that adverse childhood experiences, especially emotional neglect experiences, were related to changes in the proportion of characters, friendly interactions, and objects in adults’ dreams. The proportion of characters in the dream content was related to sleep quality, total childhood trauma scores, and emotional neglect scores. The interactions between the characters in dreams were related to the current emotional state or adversities experienced in childhood. With increasing age, sexual interactions and good fortune of the characters in dreams increased. Sexual interactions in dreams also increased with education. As the level of anxiety increased, the descriptive components of the dream reports decreased.

Although we found a correlation between sexual interaction and age and years of education, we were unable to establish an effective linear model. This result showed that the proportion of dreams containing the content of sexual interactions may increase with age and years of education, but the correlations between age, years of education, and sexual interactions in dreams may not constitute a causal relationship. Early studies found that dream content involving sexual interactions is associated with sexual fantasy, sexual daydreaming, and orgasmic experiences in real life (King et al., 2009). Inconsistent with our research, according to an extensive study, there is a negative correlation between age and sexual dreams. Although the age span (16–92 years old) was large, the study did not conduct subgroup analyses of different ages (Schredl et al., 2019). Most of the participants in our study were young college students or graduates, who sexual experiences gradually increased with age. According to the continuity assumption (Schredl and Hofmann, 2003), this may be one of the reasons why sexual contents in dreams gradually increase with age. Therefore, we assumed that the sexual content in dreams presents an inverted U-shaped change; that is, the sexual content in dreams gradually increases from adolescence but gradually decreases after middle age.

We found that as the severity of childhood adversity increased, the proportion of dreams involving characters and friendly interactions decreased, whereas the proportion of dreams involving objects increased. The correlations between these three dream content items and total childhood trauma scores were attributed to their correlations with emotional neglect. Although there has been no direct evidence prior to this study that childhood adversity affects the content of dreams in adults, the effects of childhood adversity on the onset of mental symptoms or mental illness in adults have long been demonstrated. Individuals who had experiences of being emotionally neglected or abused were more likely to have diagnoses of several Axis I and Axis II mental disorders and the onset of psychosis symptoms (Rossler et al., 2014; Mandelli et al., 2015; Taillieu et al., 2016; Nelson et al., 2017). The effects of childhood experiences on the mental state of adults may be based on irreversible changes in brain function after experiencing adversities, such as trauma. As children with self-reported childhood adversities have elevated emotional reactivity to negative stimuli (Heleniak et al., 2016), the amygdala’s response to negative stimuli is similarly elevated (McCrory et al., 2013). This elevated amygdala response may be attributed to amygdala volume changes after exposure to childhood adversities (Tottenham et al., 2010; Edmiston et al., 2011; Hanson et al., 2015). If these adversities continue to exist as chronic stress, the amygdala is hyperactivated (Rosenkranz et al., 2010). In addition to volume changes in the amygdala, increasing exposure to emotional neglect also leads to hyperactivity of the hypothalamic–pituitary–adrenal (HPA) axis and dysfunctional attitudes (Peng et al., 2014; Roth et al., 2018). Studies have shown that emotional experiences in a state of awakening are more likely to occur in dreams (Malinowski and Horton, 2014). This may reflect childhood emotional neglect playing an important role not only in psychological growth, but also in dream formation, and these experiences could influence dreams in adults.

Emotional neglect has been associated with difficulties in emotional processing and the development of secure attachment (Kessler et al., 1997). Earlier studies have shown that adversity in childhood, especially emotional neglect, is associated with the onset of anxiety disorder (SAD; Kessler et al., 1997). Greater emotional neglect predicts significantly greater severity of SAD, lower quality of life, and higher levels of self-reported disability (Bruce et al., 2012). This may explain why people with higher levels of emotional neglect have fewer characters and friendly interactions in their dreams. A history of emotional neglect has been positively associated with loneliness, and this loneliness could increase their emotional attachment to objects (Kyrios et al., 2018; Yap et al., 2020). People with higher levels of emotional neglect were less likely to stick with smaller rewards and more likely to make choices to avoid harm (De Carvalho et al., 2015). This result may support the social simulation theory of dreaming (SST; Revonsuo et al., 2016), which suggests that dream contents serve as simulations for real-life social events. This might be the reason our participants who experienced more emotional neglect had more objects in their dreams. Fewer characters, friendly interactions, and more objects in dreams reflect of the strategies adopted by people with higher levels of emotional neglect to avoid harm. Interestingly, fewer strangers and more familiar characters in dreams have been found following short social isolation (Tuominen et al., 2022).

Childhood trauma and emotional neglect can disrupt the relationships between individuals and objects. To reduce fear, disappointment, sadness, and abandonment, individuals shift their needs from human interactions to object interactions, such as eating more and satisfying material needs to meet their psychological needs. This could lead to reduced interactions with people in dreams and increased attention to objects. Similarly, as it has been illustrated that the impact of childhood adversities on psychological development extends until middle age in adults, the findings of this study show that the impact of childhood adversity on dream content can extend to adulthood as well.

Although this study provided important information on dream content associated with childhood adversities, especially emotional neglect, several limitations should be considered. First, the best study design to illustrate the relationship between childhood adversities and dream content in adults is a prospective study. Second, qualitative interviews are needed to identify the exact causality between childhood adversities and dream content. Third, the dream content written by the participants was diverse and subjective. Fourth, the sample size was relatively small, and the purpose of this study was to explore the effects of childhood trauma on dream content in healthy adults, without including a wider range of participants who had experienced childhood adversities; therefore, our study is not sufficient to represent all situations. In the future, research on the relationship between childhood experiences and dream content should be based on longitudinal cohort studies in which the effects of different adversity experiences dream content can be continuously observed. Therefore, we should also simultaneously explore the intermediary or regulatory factors between childhood adversity and dreams, and design appropriate interventions to reduce the negative effects of childhood adversity on adult dreams.

## Conclusion

Childhood adversities may influence adult dream content, and this influence may consist of the association of emotional neglect with characters, friendly interactions, and objects in dream content.

## Data Availability Statement

The raw data supporting the conclusions of this article will be made available by the authors, without undue reservation.

## Ethics Statement

The studies involving human participants were reviewed and approved by Peking University Sixth Hospital Review Board. The patients/participants provided their written informed consent to participate in this study.

## Author Contributions

YM designed the study, analyzed the data, drafted the manuscript, conducted the literature review, and revised and finalized the manuscript. YM, XF, DW, XZ, and ZY carried out the subject recruitment, questionnaire screening, dream content collection, and data entry. XZ and ZY performed the electronic entry and coding of dream content. YB, RZ, and JD guided the statistical analysis of the data and reviewed and revised the manuscript. QS helped with the literature review and revised the manuscript. LL provided the platform and support for the research and reviewed and revised the manuscript. HS supervised the study design and critically reviewed and revised the manuscript. All authors contributed to the article and approved the submitted version.

## Conflict of Interest

The authors declare that the research was conducted in the absence of any commercial or financial relationships that could be construed as a potential conflict of interest.

## Publisher’s Note

All claims expressed in this article are solely those of the authors and do not necessarily represent those of their affiliated organizations, or those of the publisher, the editors and the reviewers. Any product that may be evaluated in this article, or claim that may be made by its manufacturer, is not guaranteed or endorsed by the publisher.
